# Once-per-step control of ankle-foot prosthesis push-off work reduces effort associated with balance during walking

**DOI:** 10.1186/s12984-015-0027-3

**Published:** 2015-05-01

**Authors:** Myunghee Kim, Steven H Collins

**Affiliations:** Department of Mechanical Engineering, Carnegie Mellon University, Pittsburgh, Pennsylvania, 15213 USA; Robotics Institute, Carnegie Mellon University, Pittsburgh, Pennsylvania, 15213 USA

**Keywords:** Biomechanics, Locomotion, Robotic prosthesis, Stability, Ankle actuation

## Abstract

**Background:**

Individuals with below-knee amputation have more difficulty balancing during walking, yet few studies have explored balance enhancement through active prosthesis control. We previously used a dynamical model to show that prosthetic ankle push-off work affects both sagittal and frontal plane dynamics, and that appropriate step-by-step control of push-off work can improve stability. We hypothesized that this approach could be applied to a robotic prosthesis to partially fulfill the active balance requirements of human walking, thereby reducing balance-related activity and associated effort for the person using the device.

**Methods:**

We conducted experiments on human participants (N = 10) with simulated amputation. Prosthetic ankle push-off work was varied on each step in ways expected to either stabilize, destabilize or have no effect on balance. Average ankle push-off work, known to affect effort, was kept constant across conditions. Stabilizing controllers commanded more push-off work on steps when the mediolateral velocity of the center of mass was lower than usual at the moment of contralateral heel strike. Destabilizing controllers enforced the opposite relationship, while a neutral controller maintained constant push-off work regardless of body state. A random disturbance to landing foot angle and a cognitive distraction task were applied, further challenging participants’ balance. We measured metabolic rate, foot placement kinematics, center of pressure kinematics, distraction task performance, and user preference in each condition. We expected the stabilizing controller to reduce active control of balance and balance-related effort for the user, improving user preference.

**Results:**

The best stabilizing controller lowered metabolic rate by 5.5% (p = 0.003) and 8.5% (p = 0.02), and step width variability by 10.0% (p = 0.009) and 10.7% (p = 0.03) compared to conditions with no control and destabilizing control, respectively. Participants tended to prefer stabilizing controllers. These effects were not due to differences in average push-off work, which was unchanged across conditions, or to average gait mechanics, which were also unchanged. Instead, benefits were derived from step-by-step adjustments to prosthesis behavior in response to variations in mediolateral velocity at heel strike.

**Conclusions:**

Once-per-step control of prosthetic ankle push-off work can reduce both active control of foot placement and balance-related metabolic energy use during walking.

**Electronic supplementary material:**

The online version of this article (doi:10.1186/s12984-015-0027-3) contains supplementary material, which is available to authorized users.

## Background

People with below-knee amputation experience more falls and lower balance confidence than individuals without amputation [1]. Fall risk is more elevated for individuals who report needing to concentrate on each walking step [1], suggesting that difficulty with balance maintenance during steady gait might contribute to increased fall risk. Amputees using passive prostheses expend more metabolic energy during walking [2], which could also be partially due to increases in balance-related effort. For non-amputees, walking on uneven terrain [3] or with visual perturbations [4] challenges balance and increases metabolic energy cost. This increase in effort is often due not to changes in average gait mechanics, but rather to changes in step-by-step variations in, e.g., foot placement and associated muscle activity, used for the active control of balance [5]. For similar reasons, external stabilization can have an opposite effect [6]. Among amputees, destabilizing conditions have a much greater detrimental effect on energy cost, walking speed, and perceived effort [7], likely reflecting greater increases in balance-related effort. Such balance-related deficits contribute to reduced mobility, social activity and quality of life for people with amputation [8]. Fall avoidance and recovery training show promise for reducing fall rates among amputees [9-11], but are unlikely to reduce the effort associated with active maintenance of balance. Active prosthesis control could complement this approach; in addition to potentially further improving balance confidence and reducing fall rates, enhanced control might also reduce balance-related effort.

Active prostheses have already demonstrated improvements in other aspects of walking performance. Robotic ankle-foot prostheses have been used to reduce metabolic energy consumption during walking by producing more positive mechanical work at the ankle joint than conventional passive devices [12]. As the amount of prosthesis work produced during the end of the stance period, or ‘push-off’, increases, metabolic energy consumption can be reduced [13]. Just as average push-off work seems to affect nominal walking effort, perhaps adjustments in push-off work on each step could reduce the effort associated with recovering from small, intermittent disturbances on each step.

### Once-per-step push-off work control

**Figure 1:**
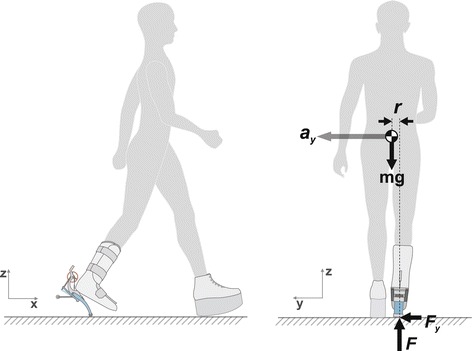
Trailing-limb push-off affects both sagittal plane and frontal plane dynamics. Ankle push-off generates a force (*F*) commonly understood to affect motions in the sagittal plane (*left*) but which also affects motions in the frontal plane (*r*ight). In general, the combination of push-off and gravity, with finite mediolateral displacement between the center of pressure and the center of mass (*r*) results in a mediolateral force at the foot (*F*_*y*_), thereby contributing to mediolateral acceleration of the body (*a*_*y*_). Neglecting rotational inertia about the center of mass, the effect on lateral acceleration is proportional to push-off force as $\Delta \textmd {\textit {a}}_{\textmd {\textit {y}}} = \frac {1}{m} \cdot \frac {\textmd {\textit {r}}}{L} \cdot \Delta \textmd {\textit {F}}$, where *L* is leg length.

Results from recent studies of walking using mathematical models and bipedal robots suggest that once-per-step control of ankle push-off work can improve balance. This approach is based on limit-cycle analysis of gait: at key moments in the gait cycle the system state is sampled, the error from the nominal state (or fixed point) for that instant is calculated, and the error is used to calculate control inputs for the ensuing step. When effective, small changes in control on each step reject small disturbances to the system, improving stability without changing the limit cycle itself. This approach has been used to stabilize two-dimensional walking robots [14] including one that set the distance record for legged robots [15]. We recently used a dynamic model of walking to investigate the effectiveness of once-per-step push-off work control at stabilizing three-dimensional bipedal gait [16], and found it to be even more effective than foot placement at recovering from random ground height disturbances. This may owe to the fact that push-off affects both frontal-plane and sagittal-plane motions (Figure 1). In three-dimensional systems, side-to-side motions tend to be less stable [17-19], making the effects of push-off on mediolateral velocity especially useful. Another advantage of ankle push-off work control for prosthesis design is that, unlike foot placement strategies, it requires actuation only at the ankle joint. Once-per-step control of ankle push-off work therefore seems like an attractive option for reducing balance-related effort for individuals with transtibial or transfemoral amputation.

Implementing a simulation-based controller in a robotic prosthesis is made challenging, however, by factors such as limited sensory information and model errors. In our simulation study, the best performance was obtained with full state feedback control, in which errors in the position and velocity of all parts of the body were used to make control decisions. This is impractical in hardware. Fortunately, we also found that mediolateral velocity measurements alone could be used to reconstruct desired ankle push-off work within 1% of the value calculated using full state feedback (Kim M, Collins SH: Once-per-step control of ankle push-off work improves balance in a three dimensional simulation of bipedal walking, in review). This reduced-order controller retained a substantial portion of the effectiveness of the full-state feedback version, and is more easily implemented in hardware.

A more significant issue is that humans are vastly more complex than the simple models used to derive candidate controllers, which could make the effects of intervention more difficult to observe. Our model included human actuation only at the hips, and treated this as independent from the behavior of the ankle-foot prosthesis [16]. In reality, we expect humans to exhibit complex, neurally-based compensation strategies throughout the body as prosthesis behavior changes, including long term adaptations. The right prosthesis behavior might still be beneficial, of course, if it were to provide a useful component of an overall coordination strategy that involves less effort by the human at steady state. Differences between prosthesis controllers might be difficult to measure, however, since the human could partially compensate for even poor control schemes. To make the effects of push-off work control on balance-related effort more obvious we simulated controllers expected to either stabilize or destabilize the user, and found the expected changes in dynamic stability of the model. A similar relationship might be expected for balance-related outcomes in humans.

Another way of magnifying the effects of prosthesis control on balance-related effort is to make balance more difficult by applying an external disturbance. Human gait exhibits some degree of variability even without explicit disturbances due to internal actuation and sensor noise [20-22]. When only small external disturbances are applied, the differences in many measures of balance-related effort can be masked by baseline noise. In our simulation model we found that low levels of ground height disturbance caused negligible changes in mechanical work requirements at the hip and ankle. A significant external disturbance can make these changes more obvious. A common disturbance encountered by individuals with amputation is ground irregularity [7]. This is difficult to implement in a laboratory setting, but a similar effect can be achieved with a robotic prosthesis by applying unexpected changes in the landing angle of the foot at heel strike. This affects the ensuing collision, resulting in significant changes in system energy and both fore-aft and lateral components of center of mass velocity (similar to the effect of push-off illustrated in Figure 1). Such a disturbance would therefore be expected to increase active control requirements and balance-related effort.

### Measuring balance-related effort

Differences in balance-related effort across prosthesis controllers could be indicated by a combination of step width variability, average step width, within-step center of pressure variability, metabolic rate, cognitive load or user preference. In the present context, ‘balance-related effort’ refers to the portion of activity associated with balance maintenance during walking, as opposed to activity for ‘propulsion’, ‘body weight support’, or other nominal gait requirements. Such effort can be isolated from nominal walking effort if changes are made only in step-by-step prosthesis dynamics, associated with balance, and not to average prosthesis mechanics. Even if the human user were to adjust their average gait mechanics in response to such prosthesis control, for example by taking wider or narrower steps, such changes would primarily relate to changes in balancing strategy and not to the nominal effects of the device.

Step width variability is an indicator of effort arising from active control of foot placement. Subjects tend to increase step width variability in the presence of a disturbance [3,4,22] and decrease variability with external stabilization [18,23]. This suggests increased or decreased use of foot placement control, and associated effort, when balance is challenged or assisted, respectively. If prosthetic ankle push-off control were to make balancing easier for the human user, we might therefore expect to observe reduced active control of step width and reduced variability.

Increased average step width can also indicate an increase in balance-related effort. Humans sometimes increase step width when balance is challenged through sensory-motor impairment [6,24] or external disturbances [3]. This strategy, perhaps used to increase ‘margin of stability’ [25], comes at the cost of increased metabolic energy consumption, which increases with the square of step width [26]. Our recent simulation study also showed that increasing step width enhanced stability but increased energy cost. If prosthesis push-off control were to reduce the need for active balance, this might therefore lead to reduced step width and lower metabolic rate.

Center of pressure variability within the stance phase of each step might also reflect changes in balance-related effort. Strategies based around within-step center of pressure control, including ‘zero moment point’ control, are widely used to stabilize walking robots [27]. In the presence of disturbances to ground height, the center of pressure can be continuously controlled by the ankle joint to maintain balance [28]. In our recent simulation study, we found that ankle inversion-eversion torque control could stabilize gait, resulting in a small (about 1%) increase in center of pressure variability. Larger center of pressure variability in the intact limb of individuals with transfemoral amputation suggests that this strategy may be utilized more heavily when other balance pathways are impaired [29]. With improved prosthesis control, we might expect to find small reductions in center of pressure variability for the intact foot.

Changes in metabolic energy consumption can capture the overall effects of altered muscle activity associated with balance. When people are exposed to significant, random disturbances during gait, their metabolic energy consumption can increase by up to 27% [3,4,7]. Conversely, providing external stabilization can reduce energy cost by up to 8% [18,23]. Such changes are often not associated with altered nominal gait patterns, but rather with step-by-step adjustments in gait mechanics, apparently indicating changes in step-by-step muscular effort associated with balance [3]. If prosthesis push-off control were to supplant a portion of the human user’s balance-related effort, we would expect a reduction in metabolic energy consumption.

Walking seems to require the use of some cognitive resources [30] and humans appear to divide available resources between walking and other simultaneous tasks [31,32]. Individuals with sensory-motor deficits have been observed to sacrifice performance at secondary tasks in an attempt to maintain low gait variability [31], while fall-prone individuals have been observed to pay an energetic penalty (by taking wider steps) so as to maintain both distraction task performance and low gait variability [32]. An effective ankle prosthesis controller may therefore result in either improved performance at distraction tasks or greater improvements in other outcomes under distraction-task conditions.

User preference is arguably the most important measure of prosthesis performance, and it strongly correlates with positive reception of a device by consumers [33]. Individuals with amputation strongly desire prostheses that positively impact balance [34,35], and prefer actively-controlled prosthetic knees [36,37] that reduce fall likelihood [38]. All other things being equal, we would therefore expect users to prefer prosthesis controllers that contribute to balance maintenance.

### Study aims and hypotheses

The goal of this experiment was to examine the effects of once-per-step modulation of prosthetic ankle push-off work on balance-related effort. We hypothesized that appropriate control of ankle push-off work would reduce the effort required to maintain balance during walking, which would be indicated by improvements in some combination of step-width variability, average step width, within-step center of pressure variability, metabolic rate, distraction task performance, and user preference. We hypothesized that an inverse controller would destabilize the user, leading to a deterioration in the same outcome measures. We also tested two baseline conditions, walking in street shoes and walking in the prosthesis simulator without external disturbances, to verify that the use of the prosthesis and the application of external disturbances each increased balance-related effort. We expected the results of this study to inform follow-up experiments among individuals with amputation, eventually leading to the design of prosthetic limbs that reduce balance-related effort during walking.

## Methods

We performed an experiment to investigate how once-per-step control of ankle push-off work affects balance-related effort. We developed a discrete ankle push-off work controller based on a mathematical model (Kim M, Collins SH: Once-per-step control of ankle push-off work improves balance in a three dimensional simulation of bipedal walking, submitted) and implemented it on an existing robotic prosthesis emulator [39] worn by non-amputees using a simulator boot. We conducted a walking experiment with a variety of controllers expected to stabilize, destabilize, or have no effect on the user, while maintaining constant average mechanics. We increased initial balance-related effort by applying a random disturbance to the landing angle of the prosthetic foot and having subjects complete a cognitive distraction task. We also collected two baseline conditions, one with no landing-angle disturbance and the other without the prosthesis. We measured step width variability, average step width, within-step center of pressure variability, metabolic energy consumption, distraction task accuracy, and user preference as indicators of balance-related effort.

### Prosthesis control

#### Hardware platform

**Figure 2:**
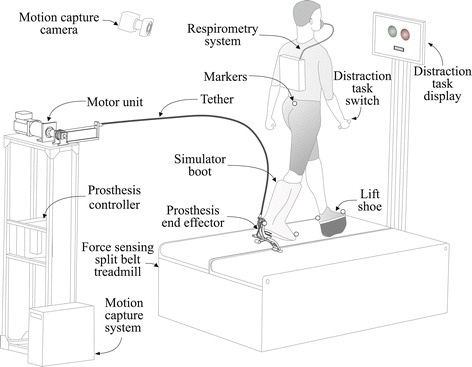
Experimental setup. Subjects wore an ankle-foot prosthesis emulator on one leg using an amputation-simulating boot while walking on a force-sensing split-belt treadmill. The prosthesis system was composed of a lightweight prosthesis end-effector, a Bowden cable tether, and a powerful off-board motor and controller. On the opposite limb, subjects wore a lift shoe with a rocker bottom. Reflective markers were attached to the sacrum and the toe and heel of each foot. Marker data was both streamed to a real-time controller and logged by a motion capture system. Subjects wore a wireless respirometry system to measure metabolic rate. Subjects completed a distraction task in which they observed patterns of colors on a monitor and provided responses using a hand-held switch.

We used a tethered, one degree of freedom, ankle-foot prosthesis to implement once-per-step ankle push-off work control. This platform (Figure 2, described in detail in [39]) used series elastic actuation and had peak operating torque of 175 N ·m, root-mean-squared torque tracking error of 3.7 N ·m, peak joint power of 1.0 kW, closed-loop torque bandwidth of 17 Hz and prosthesis end-effector mass of 0.96 kg. The system was actuated by a large offboard servomotor and controlled by a high-bandwidth real-time computer (ACE1103, dSPACE Inc., Wixom, MI). Prosthetic ankle angle and torque were measured using onboard sensors.

Mediolateral velocity of the body was measured online using a marker-based motion capture system. A 7-camera system (Vicon, Oxford, UK) measured the position of a reflective marker attached at the sacrum (Figure 2), sampled at a rate of 100 Hz. Lateral velocity of the sacral marker, calculated as the time derivative of sacral marker position, was used to approximate lateral velocity of the center of mass.

Foot contact was determined online using a split-belt treadmill with six-axis force sensing (Bertec Co., Columbus, OH, USA). The sampling rate was 1000 Hz, and data were low-pass filtered at 100 Hz to reduce noise. Foot contact was detected when the vertical component of force was above a threshold value of 20 N. This removed unreliable center of pressure measurements during periods of low force, such as during initial heel contact and just prior to toe off, which could cause artificially high variations in the center of pressure.

#### Controller design

We implemented once-per-step control of ankle push-off work using mediolateral velocity as a reference. The controller was composed of a high-level discrete controller and a low-level continuous controller.

**Figure 3:**
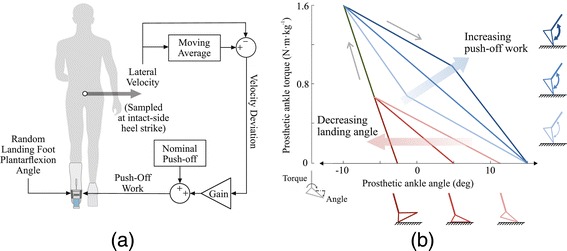
Control architecture.**(a)** The high-level controller determined desired ankle push-off work based on mediolateral velocity once per step. Desired push-off work was calculated at the instant of intact-side heel strike, and was equal to a nominal value plus the product of a gain and the difference between lateral velocity on that step and the average lateral velocity over the prior ten steps. Landing-angle disturbances were randomly selected at the beginning of each swing phase. **(b)** The low-level controller continuously regulated ankle torque within each step according to a desired torque-angle relationship. The torque-angle curve was updated by the high-level controller on each step, reflecting changes in desired push-off work (blue portion) and landing-angle disturbance (red portion).

The high-level controller made adjustments once per step that were intended to stabilize or destabilize the user’s gait (Figure 3(a)). We calculated the desired magnitude of ankle push-off work as a linear function of the error between nominal lateral velocity and measured lateral velocity, sampled at the moment that the heel of the intact-side foot touched the ground:

(1) $$ W_{des} = W_{des}^{*} + K \cdot \left(v_{ref} - v_{ml}\right)  $$

where *W*_*des*_ is the desired ankle push-off work for this step, $W_{\textit {des}}^{*}$ is the nominal desired push-off work (approximately equal to the average work over many steps), *K* is the high-level control gain (with positive values expected to contribute to balance), *v*_*ml*_ is the lateral velocity of the sacral marker on this step, and *v*_*ref*_ is the reference lateral velocity calculated as a moving average over ten steps (used to prevent changes in average mechanics from affecting balance-related prosthesis control). During pilot tests, we found that not all subjects preferred the same gains, and so we used two magnitudes that seemed to span the most effective range (0.4 and 0.8).

The low-level controller continuously regulated ankle torque as a function of ankle angle so as to deliver the desired magnitude of push-off work over the course of a step, as described in detail in [13]. Desired ankle torque was calculated as a piece-wise linear function of ankle angle, with separate paths for dorsiflexion and plantarflexion phases (Figure 3(b)). On each step, the plantarflexion portion of this curve was altered so as to generate the desired magnitude of net push-off work determined by the high-level controller. The plantarflexion torque-angle curve was also adjusted to accommodate differences in peak dorsiflexion angle on each step. The torque control layer then tracked desired torque by rotating an off-board motor [39]. During the swing phase, the low-level controller performed position control.

#### Disturbances

We applied a disturbance in the form of a landing foot angle that was randomly changed on each step. Landing angle was defined as the plantarflexion angle of the prosthesis toe at the moment of foot contact with the ground (Figure 3(b)). Landing angle for the next step was randomly selected at the moment the toe lifted off the ground, and the toe was servoed to this configuration during swing. Because of the low inertia of the toe [39] and the cushioning effects of the simulator boot, subjects could not sense differences in toe positioning during swing. Toe angle was maintained until just after the prosthesis toe contacted the ground, as sensed by a spike in ankle torque, at which time the prosthesis switched back into torque control mode. During the ensuing stance phase, the plantarflexion portion of the desired torque-angle curve was adjusted such that the disturbance itself had no effect on net prosthesis work.

### Experimental methods

#### Subjects

Walking experiments were conducted with able-bodied adults (N = 10 [9 male and 1 female], age = 25 ±4.8 yrs, body mass = 81.2 ± 5.8 kg, leg length = 0.99 ± 0.03 m, mean ± s.d.). Leg length was defined as the distance between markers at the heel and sacrum. To simulate the effects of amputation, subjects wore the prosthesis using a simulator boot and wore a lift shoe on the other leg (Figure 2). All participants had prior experience using the prosthesis emulator. All subjects provided written informed consent prior to participating in the study, which was conducted in accordance with a protocol approved by the Carnegie Mellon University Institutional Review Board (HS13-444).

#### Experimental protocol

**Figure 4:**
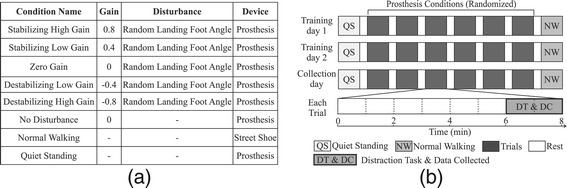
Experimental protocol.**(a)** Each day of the experiment included eight conditions, five of which compared high-level control gains and three of which provided baseline data. During all controller conditions, a disturbance was applied in the form of randomly-changing landing foot angle. In the No Disturbance baseline condition, the high-level gain was set to zero and the disturbance was not applied. In the Normal Walking baseline condition, subjects walked in street shoes without the prosthesis. In the Quiet Standing baseline condition, subjects stood still while wearing the prosthesis. **(b)** Each subject participated in two training days followed by a collection day. Each day, subjects were presented with Quiet Standing, followed by the six prosthesis conditions in random order, and finally the Normal Walking condition. Subjects walked for eight minutes in each trial, followed by three minutes of rest. During minutes six through eight, subjects completed the distraction task. All results presented in the main text are from data collected in minutes six through eight of each trial on the third day.

Subjects experienced eight conditions per collection (Figure 4(a)). Five conditions compared once-per-step push-off work controllers with gains of 0.8, 0.4, 0, -0.4 and -0.8, labeled Stabilizing High Gain, Stabilizing Low Gain, Zero Gain, Destabilizing Low Gain, and Destabilizing High Gain conditions, respectively. The Stabilizing conditions were expected to reduce balance-related effort and the Destabilizing conditions were expected to increase balance-related effort compared to the Zero Gain condition. Landing-angle disturbances were applied in all five of these conditions. Two additional walking conditions provided baseline data. Data were collected for Normal Walking in street shoes and for a No Disturbance condition in which the prosthesis did not apply the landing-angle disturbance. These baseline conditions allowed evaluation of the effects of wearing the prosthesis and applying the disturbance on balance-related effort. Finally, a Quiet Standing condition in which subjects stood still while wearing the prosthesis allowed measurement of resting metabolic rate.

Subjects walked for eight minutes in each walking trial, with three minutes of rest between each (Figure 4(b)). A distraction task was performed during the sixth through eighth minutes of each walking trial. Subjects performed all trials in random order, except for Quiet Standing, which was always performed first, and Normal Walking, which was always performed last. Subjects experienced all eight conditions three times on separate days, the first two of which were used for training. All data presented here are from the collection on the third day.

#### Measures of balance-related effort

We measured metabolic energy consumption, step width variability, average step width, within-step center of pressure variability, distraction task error rate, and user preference. Data were collected during the final two minutes of each trial.

Metabolic energy consumption was obtained through indirect calorimetry using a wireless breath-by-breath respirometry system (Oxycon Mobile, CareFusion, San Diego, CA, USA). Subjects fasted for at least four hours prior to each collection. The rate of oxygen consumption and carbon dioxide production were recorded, and the last two minutes of data were averaged. Steady state oxygen consumption was confirmed by visual inspection. Metabolic rate was calculated using a standard equation [40] and normalized to body mass. The value for Quiet Standing was subtracted to obtain net metabolic rate.

Step width variability and average step width were calculated using both foot markers and center of pressure data. Step width was defined as the mediolateral displacement between consecutive foot positions. Foot locations were determined at mid-stance, defined as the moment when the sacral marker was directly above the heel marker in the sagittal plane. Marker data and center of pressure data were first low-pass filtered with a cutoff frequency of 20 Hz. We then used the average of the locations of the toe and heel markers at mid-stance to determine marker-based foot position [22] and center of pressure location at mid-stance to determine center-of-pressure-based foot position [18]. Average step width and step width variability were calculated as the mean and standard deviation, respectively, of all step widths in the corresponding two-minute period.

Within-step center of pressure variability was calculated as the standard deviation of the mediolateral location of the center of pressure at each instant in the stance period. The average center of pressure was subtracted for each step, and center of pressure trajectories were normalized in time to percent stance. At each instant of stance, the standard deviation of center of pressure location across steps was calculated. These values were then averaged across all instants in stance. Center of pressure measurements during initial foot contact or just before toe off are unreliable, but were not included because stance was defined as the period for which the vertical component of the ground reaction force was above a threshold.

Cognitive load was probed by measuring accuracy at a vision-based distraction task for two minutes at the end of each trial. A pair of circles having either the same color (both red or both green) or different colors (red and green or *vice versa*) were shown on a screen (Figure 2) every two seconds. Subjects were instructed to press a hand-held button when two consecutive pairs of circles had the same pattern, *i.e.* same followed by same or different followed by different. Error rate was calculated as the percentage of incorrect responses. All subjects reported an ability to distinguish between circle colors. One subject had error rates more than three standard deviations outside the mean, likely resulting from a misunderstanding of the instructions, and their task performance data were removed from the study.

User preference was obtained by asking subjects to rate each condition on a numerical scale. Normal Walking was used as the reference at zero, with -10 corresponding to “unable to walk” and +10 corresponding to “walking is effortless”. Ratings were performed immediately following each walking trial.

A video showing a typical experimental session, including prosthesis hardware and the distraction task, is provided as Additional file 1.

#### Statistical analysis

We first investigated whether different control gains had any effect on each outcome using repeated measures ANOVA with significance level *α* = 0.05. In cases where significant effects were found, we compared each of the five controller conditions using paired t-tests. We also performed paired t-tests comparing Normal Walking and No Disturbance conditions, to test for an effect of wearing the prosthesis, and between the No Disturbance and Zero Gain conditions, to test for an effect of the disturbance.

## Results

Stabilizing and destabilizing controllers modulated ankle push-off work on each step while maintaining consistent average push-off work. Metabolic energy consumption and step width variability were lower in Stabilizing conditions compared to Zero Gain or Destabilizing conditions. Control gain did not have a statistically significant effect on other balance-related outcomes, but users appeared to prefer Stabilizing conditions. Wearing the prosthesis increased metabolic rate and decreased user preference compared to Normal Walking. The landing-angle disturbance further increased metabolic rate and decreased preference, and also appeared to increase step width variability.

### Prosthesis mechanics

**Figure 5:**
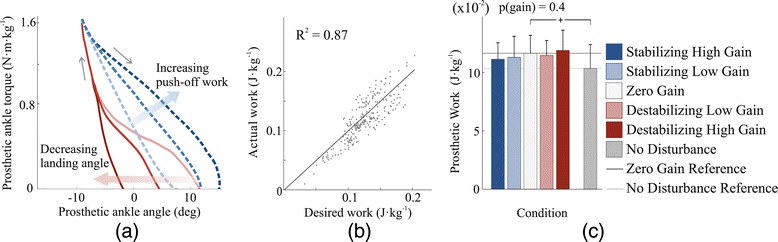
Ankle-foot prosthesis mechanics.**(a)** Measured torque-angle relationships for three landing angles and three push-off work values. The red solid lines show the average of all steps in which landing angle was less than 1° (dark line), between 5° and 7° (medium line), and greater than 9° (light line). The blue dashed lines show the average of all steps in which net ankle push-off work was less than 1.3 times the value in Normal Walking (light line), between 1.8 and 2.3 times normal (medium line), and at least 2.8 times normal (dark line). **(b)** The low-level controller closely tracked the desired angle-torque curve, resulting in a strong correlation between desired and measured ankle push-off work on each step. Data are shown for a representative trial. **(c)** Average push-off work remained within 5% of the value for the Zero Gain condition across all other control gains. Subjects received slightly less energy per step in the No Disturbance baseline condition. Blue bars correspond to Stabilizing Gain conditions, white bars to the Zero Gain condition, and red bars to Destabilizing Gain conditions. Darker blue and red bars correspond to High Gains. Light gray bars correspond to the No Disturbance condition. The p-value at top is for a repeated measures ANOVA test for an effect of control gain. Pluses (+) indicate statistical significance among baseline conditions.

The prosthesis applied landing-angle disturbances and modulated ankle push-off work as desired on each step. Landing angles ranged from −3° to 12° of plantarflextion across steps (Figure 5(a), solid lines). Net push-off work ranged from 0.00 to 0.34 J ·*kg*^−1^ across individual steps, as commanded by the controller (Figure 5(a), dashed lines). Desired ankle torque was tracked with root-mean-squared error of 7% across all subjects and conditions, resulting in strong correlation between desired and measured net ankle push-off work across individual steps (R ^2^ = 0.87, Figure 5(b)).

Average push-off work did not change significantly across controller conditions (p = 0.4). Average net prosthesis work remained within 5% of the value in the Zero Gain condition for all other controller conditions (Figure 5(c)). Average prosthesis push-off work appeared to be slightly lower in the Stabilizing control conditions than in the Zero Gain condition.

### Metabolic rate

**Figure 6:**
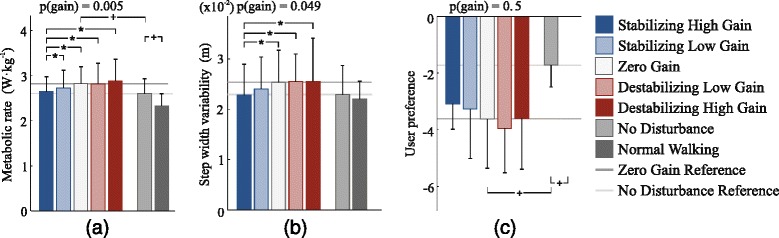
Balance-related outcomes.**(a)** Metabolic rate was reduced with Stabilizing control compared to Zero Gain and Destabilizing control conditions. For example, metabolic rate was 8.5% lower in the Stabilizing High Gain control condition than in the Destabilizing High Gain control condition (p = 0.02). Wearing the prosthesis increased metabolic rate, as did application of the disturbance. **(b)** Step width variability was lower with Stabilizing control than in Zero Gain or Destabilizing Gain conditions. Wearing the prosthesis appeared to increase step width variability, as did application of the disturbance. **(c)** Subjects appeared to prefer Stabilizing control conditions, although this trend was not statistically significant. Subjects preferred Normal Walking over wearing the prosthesis, and preferred not to have the random landing-angle disturbance. Blue bars correspond to Stabilizing control conditions, white bars to the Zero Gain condition, and red bars to Destabilizing conditions. Darker blue and red bars correspond to High Gains. Light gray bars correspond to the No Disturbance condition, and dark gray bars correspond to the Normal Walking condition. The p-values at top are for repeated measures ANOVA tests for an effect of control gain. Asterisks (*) indicate statistical significance among control gain conditions, and pluses (+) indicate statistical significance among baseline conditions.

Control gain significantly affected metabolic rate (ANOVA, p = 0.005), with Stabilizing controllers leading to decreased metabolic energy consumption. The Stabilizing High Gain controller reduced metabolic energy consumption compared to all other gains (p ≤ 0.04; Figure 6(a)), including a 5.5% reduction compared to the Zero Gain condition (p = 0.003) and an 8.5% reduction compared to the Destabilizing High Gain condition (p = 0.02).

Random landing-angle disturbances increased metabolic rate by 9.0%, compared to the No Disturbance condition (p = 0.02). Normal Walking required 10.4% less metabolic energy than the No Disturbance condition (p = 0.0008).

### Step width variability

Variability in step width as measured by center of pressure was affected by control gain (ANOVA, p = 0.049), with Stabilizing controllers leading to reduced variability. Stabilizing High Gain control reduced step-width variability by 10.0%, 10.5%, and 10.7% compared to Zero Gain, Destabilizing Low Gain, and Destabilizing High Gain conditions, respectively (p = 0.009, 0.046, and 0.030; Figure 6(b)). A similar result was observed for step width variability as measured using marker information (Additional file 2: Figure A1).

The random landing-angle disturbance (Zero Gain condition) appeared to increase step width variability by about 10% compared to the No Disturbance condition, but this trend was not statistically significant (p = 0.2; Figure 6(b)). Walking with the prosthesis in the No Disturbance condition did not increase step width variability compared to Normal Walking (p = 0.6).

### User preference

Users appeared to prefer Stabilizing control conditions over Zero Gain and Destabilizing control conditions, but this trend was not statistically significant (ANOVA, p = 0.5; Figure 6(c)). Applying the random landing-angle disturbance (Zero Gain condition) substantially reduced user preference compared to the No Disturbance condition (p = 0.001). Subjects preferred the Normal Walking condition over all other conditions (p ≤ 0.007).

### Other outcomes

Within-step center of pressure variability seemed to be reduced by Stabilizing controllers, but this trend was not statistically significant (ANOVA, p = 0.3). Wearing the prosthesis appeared to increase within-step center of pressure variability by 14% compared to Normal Walking, and the landing-angle disturbance appeared to increase within-step center of pressure variability by an additional 10%, but neither of these changes were statistically significant (p = 0.08 and p = 0.1).

Average step width, average stance period and average stride period were unchanged across controller conditions (less than 1.2% change; ANOVA, p ≥ 0.1). Wearing the prosthesis increased average step width by 30% compared to Normal Walking (p = 5 ·10^−7^), and the landing-angle disturbance increased average step width by an additional 6% (p = 0.009) as measured using foot markers, with similar results using center of pressure (Additional file 2: Figure A1).

The rate at which subjects made errors in response to the distraction task was unchanged across controller conditions (ANOVA, p = 0.3).

Complete results, including means, standard deviations, and statistical outcomes for all metrics, can be found in the Additional file 2: Figure A1 and Tables A1–A5.

## Discussion

We investigated the effects of once-per-step control of prosthetic ankle push-off work on balance-related effort among non-amputees walking with a prosthesis simulator. We hypothesized that controllers that appropriately modulated push-off work would reduce balance-related effort, while controllers with the opposite effect would increase effort. We found that stabilizing controllers decreased metabolic energy consumption and step width variability, while destabilizing controllers tended to have the opposite effect. Changes were not due to average push-off work or average gait mechanics, which were unchanged across controller conditions. This provides strong evidence that discrete control of prosthesis push-off work can contribute to balance during walking, reducing the need for other balancing strategies such as foot placement, and thereby reducing overall effort.

The primary link between changes in metabolic rate and underlying mechanics seems to be through variability in foot placement. We previously found that once-per-step control of push-off work was effective at stabilizing lateral motions in a three-dimensional model of gait, reducing the need for active control of foot placement [16]. With stabilizing prosthesis control, subjects may have been able to allow more natural leg swing motions, with less need for postural adjustments at heel strike, explaining the observed reductions in foot placement variability. Reduced activity in hip adductors and abductors, implicated in other studies in which balance was made easier or more difficult [3,4,18], might account for the observed reduction in metabolic rate. The muscular origins of altered balance-related effort with these controllers could be explored further by collecting electromyographic data in future studies.

Changes in average prosthesis behavior could also affect metabolic rate, but do not seem to be responsible for the changes observed in this study. Average ankle push-off work can have a substantial effect on metabolic rate [13]. To avoid confounding balance-related outcomes, we designed the prosthesis controller to have consistent average push-off work regardless of once-per-step control gain. Average push-off work was thereby held within 5% of the value in the Zero Gain condition for all Stabilizing or Destabilizing control conditions. This is a small difference compared to the step-by-step variations in push-off work, which deviated from the average by more than 100% on some steps (Figure 5(b)). Stabilizing High Gain control resulted in the lowest metabolic rate but also the lowest average push-off work. Based on a previously established empirical relationship [13], we would have expected this small change in average work to result in a 1% increase in metabolic rate rather than the 5.5% decrease we observed. It is therefore possible that more consistent average push-off work would have further enhanced the benefits of stabilizing control. Subjects also did not change their average step length or step width across controllers, which could otherwise have affected metabolic rate [26,41]. The observed reductions in metabolic rate, as with step width variability, are therefore best explained by differences in the way push-off work was varied on a step-by-step basis and the effects of such control on balance-related effort for the human.

Changes within baseline conditions also provide insights into the relationships between the use of a prosthesis, external disturbances and balance-related effort. Compared to Normal Walking, simply wearing the prosthesis had a detrimental effect on metabolic rate, average step width, within-step center of pressure variability, and user preference. Some portion of these changes may be due to, *e.g.*, the added mass, height and bulk of the prosthesis simulator boot, but some are likely indicative of increases in balance-related effort from prosthesis use. The addition of a disturbance in landing angle further worsened metabolic rate, average step width and user preference. This suggests that the landing-angle disturbance was effective at increasing balance-related effort, and may have implications for the effects of unpredictable terrain on balance-related effort for individuals with amputation. We separately tested the effect of random changes in push-off work, rather than landing angle, on balance-related effort (Additional file 2: Figure A3), and found that it similarly increased metabolic rate and other indicators of active balance. This provides further support for the idea that step-by-step changes in ankle push-off strongly affect balance.

Pair-wise comparisons of changes in metabolic rate and step width variability did not always yield statistical significance, but our confidence in the reported findings is bolstered by the consistency of the observed changes. Subject-averaged metabolic rate was lower in all Stabilizing control conditions than in the Zero Gain condition, which in turn was lower than in all Destabilizing control conditions. Subject-averaged step width variability, as measured either by center of pressure or marker data, was lower in the Stabilizing High Gain control condition than in all Zero Gain and Destabilizing gain conditions. To further test these relationships, we also examined metabolics and step width variability data from the two minutes before the distraction task was applied, and found the same stratification (Additional file 2: Figure A2(a-c)). The one finding inconsistent with our expectations was that Destabilizing High Gain control appeared to result in reduced step width variability compared to Zero Gain conditions in some cases. This was not consistent with changes in metabolic rate, but was echoed by a trend in user preference. It might be that participants adjusted their balancing strategy in the presence of larger disturbances in ways that were not fully captured by the measures used here. Nevertheless, changes in metabolic rate and step width variability consistently favored the hypothesized effects of push-off control on balance-related effort.

We did not observe statistically-significant changes in mean step width, within-step center of pressure variability, error rates at the distraction task, or user preference across control gains. In some cases, such as with user preference and within-step center of pressure variability, there appeared to be trends resembling those observed in metabolic rate and step width variability, but they were not statistically significant. A greater number of subjects would have allowed validation or rejection of these trends (post-hoc power analyses suggest that an additional forty subjects would have been needed). In other cases, such as with average step width, there were no apparent trends. It may be that subjects relied heavily on foot placement and inversion-eversion control in this task, rather than utilizing a greater margin of stability. The lack of a trend in distraction task error rate is most likely due to a poorly-calibrated task; subjects were approximately 97% accurate in all conditions. Future investigations of cognitive load under similar conditions would lend more insight if they involved a more challenging distraction task.

We did not consider trunk and arm motions in this study, which could have provided an additional resource for balance. Evidence for stabilization strategies using the trunk and arms have been observed in human walking [42,43], and variabilities of related measures have been suggested as indicators of stability [44,45]. Increased balance-related effort in the arms and trunk might explain increases in metabolic rate despite apparent reductions in step width variability observed in the condition with Destabilizing High Gain control.

We did not have a hypothesis as to which stabilizing control gain would result in greater reductions in balance-related effort, but the observed benefits of the high-gain controller might be explained by subject adaptation. In pilot tests, we observed that subjects with more experience tended to prefer higher gains for the stabilizing controller. We chose two gains that seemed to span the range preferred by both novice and trained users so as to demonstrate some benefit even if little learning occurred. It may be that, by the end of the third day of the experiment, subjects had learned how to best use the stabilizing controller and therefore saw more benefit in the higher gain condition. It is possible that an even higher gain on this feedback loop would have provided experienced subjects with greater reductions in balance-related effort.

Applying the ground disturbance through landing angle of the prosthetic foot was effective in this case, but is not ideal. If there were intrinsic coupling between prosthesis actions related to disturbance and those related to recovery, this could have made balance maintenance easier or more difficult among all control gains. Such a possibility is mitigated by the fact that the disturbance was applied early in the stance phase while stabilizing control actions were performed late in stance. More reassuring is that the disturbance was applied randomly, while once-per-step control was deterministic, meaning that any interactions were likely to wash out over the hundreds of steps measured during the trial. Another concern was the possibility that subjects might predict landing angle based on proprioception. Fortunately, subjects reported that they could not anticipate disturbances, which is supported by increases in balance-related effort when the disturbance was applied. Nonetheless, applying a fully external ground disturbance would avoid the possibility of such interactions and predictions.

Further study will be required to test whether these results are applicable to individuals with amputation. The differences between amputees and non-amputees wearing a simulator boot are numerous, including different levels of training with prostheses and the absence or presence of various sensory and motor control pathways. Perhaps for such reasons, we have previously observed opposite responses to intervention between these populations [46,47]. Less concerning are the effects of the mass, height and alignment of the prosthesis simulator, since such factors were constant across conditions and are unlikely to interact with once-per-step control gains. While the present results are promising, experiments among individuals with amputation are needed before drawing strong conclusions about effects for this population. Still, with better tuning and more sophisticated control strategies, such as regulation of both lateral and fore-aft body states, such experiments might reveal greater reductions in balance-related effort than observed here.

## Conclusions

We have demonstrated a technique for controlling prosthetic ankle push-off work once per step that reduces balance-related effort during walking in the presence of disturbances. The approach reduces metabolic energy consumption, apparently due to reductions in muscular effort associated with mediolateral foot placement. With small changes, similar control strategies could be implemented in commercially available robotic ankle-foot prostheses. Future work should investigate whether this approach provides similar improvements in balance-related effort for individuals with amputation.

## Additional files

Additional file 1
**Video of a representative data collection.** In this video, a subject walks on the instrumented treadmill with the Stabilizing High controller while performing the distraction task.

Additional file 2
**Complete data set and supplementary data.** Section 1 and Figure A1 graphically presents all data from the primary study not shown in figures in the main text. Section 2 describes a secondary analysis performed on data from minutes four to six, prior to application of the distraction task, and Figure A2 graphically presents the results from this secondary study. Section 3 describes an additional baseline condition in which push-off work was changed randomly on each step, and Figure A3 graphically presents the results from this additional baseline condition. Section 4 and Figure A4 provide prosthesis mechanics results for the additional analyses and baseline conditions. Table A1 provides mean values for all outcomes in all conditions, and Table A2 provides standard deviations for all outcomes in all conditions. Table A3 provides the results of ANOVA tests for an effect of control gain on each outcome. Table A4 provides the results of paired t-tests comparing control gain conditions, for significantly-affected outcomes. Table A5 provides the results of paired t-tests comparing baseline conditions.
