# Supplementary material for: Once-per-step control of ankle-foot prosthesis push-off work reduces effort associated with balance during walking
Source: J Neuroeng Rehabil. 2015 May 1;12:43. doi: 10.1186/s12984-015-0027-3 (PMC4429504; doi:10.1186/s12984-015-0027-3)
Supplement: Additional file 2 — Complete data set and supplementary data. Section 1 and Figure A1 graphically presents all data from the primary study not shown in figures in the main text. Section 2 describes a secondary analysis performed on data from minutes four to six, prior to application of the distraction task, and Figure A2 graphically presents the results from this secondary study. Section 3 describes an additional baseline condition in which push-off work was changed randomly on each step, and Figure A3 graphically presents the results from this additional baseline condition. Section 4 and Figure A4 provide prosthesis mechanics results for the additional analyses and baseline conditions. Table A1 provides mean values for all outcomes in all conditions, and Table A2 provides standard deviations for all outcomes in all conditions. Table A3 provides the results of ANOVA tests for an effect of control gain on each outcome. Table A4 provides the results of paired t-tests comparing control gain conditions, for significantly-affected outcomes. Table A5 provides the results of paired t-tests comparing baseline conditions. [file 12984_2015_27_MOESM2_ESM.pdf]

Additional File 2

1. Additional measures of balance-related effort

Here we graphically present the measures of balance-related effort that were not included as figures in the main text (Fig. A1). In particular, step width variability measured using marker data (rather than center of pressure as shown in the main text) was affected by control gain (ANOVA, p = 0.03), with stabilizing control resulting in reduced variability. Step width variability was 10% lower in the Stabilizing High Gain condition than in the Zero Gain condition (p = 0.03) and 12% lower than in the Destabilizing Low Gain condition (p = 0.02).

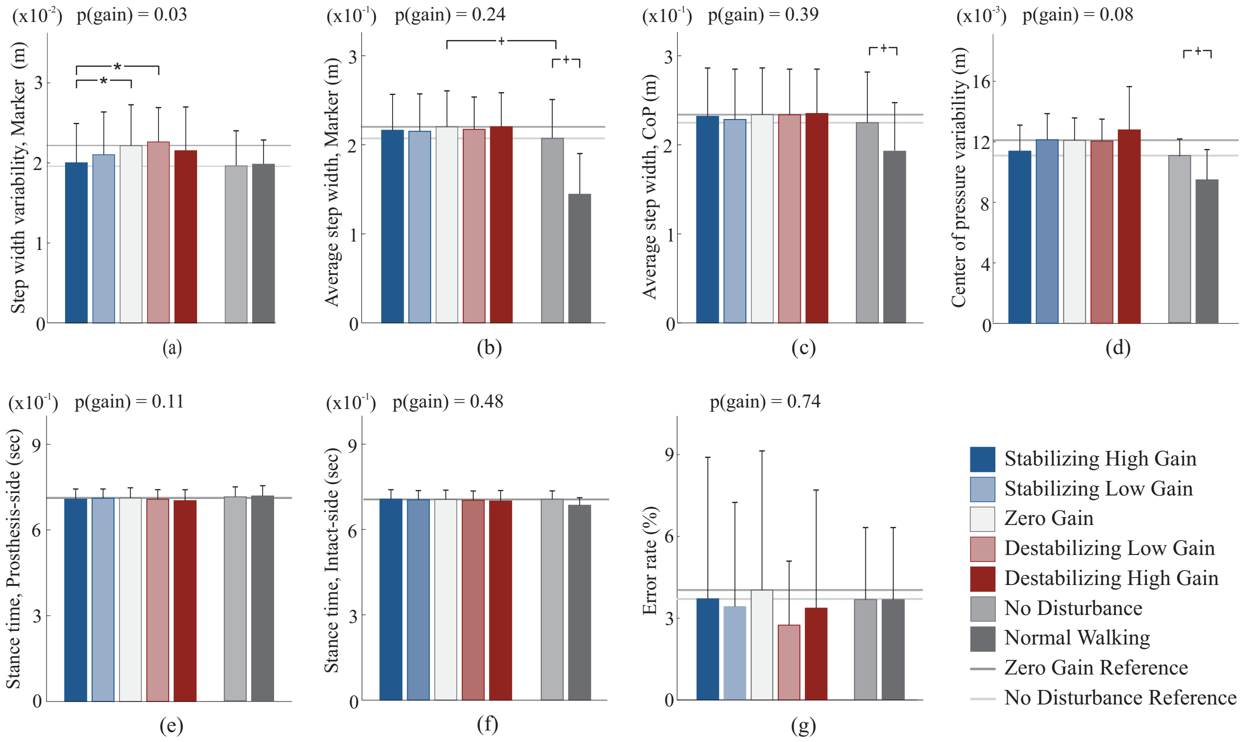

Figure A1: **Additional measures of balance-related effort.** (**a**) Step width variability based on foot markers tracked by a camera-based motion capture system decreased with Stabilizing control. (**b**) Average step width based on foot markers was greater with the prosthesis and with the disturbance. (**c**) Average step width based on Center of Pressure (CoP) measured using an instrumented treadmill was similarly affected. (**d**) Center of Pressure variability within steps seemed to be reduced with Stabilizing control. (**e**) Prosthesis-side stance time was unchanged across all conditions. (**f**) Intact-side stance time was unchanged across all conditions. (**g**) Error rate for the distraction task was unchanged across all conditions. Blue bars correspond to Stabilizing control conditions, white bars to the Zero Gain condition, and red bars to Destabilizing conditions. Darker blue and red bars correspond to High Gains. Light gray bars correspond to the No Disturbance condition, and dark gray bars correspond to the Normal Walking condition. Asterisks (*) indicate statistical significance among control gain conditions, and pluses (+) indicate statistical significance among baseline conditions.

2. Balance-related effort measured before application of the distraction task

We also analyzed balance-related measures from data taken during minutes four to six of each trial (see cf. Fig. 4 for the trial structure), prior to application of the distraction task (Fig. A2). We found similar results to those with the distraction task, reported in the main text. Metabolic energy use without the distraction task was affected by control gain (ANOVA, p = 0.001), with Stabilizing conditions leading to lower metabolic rate. For example, metabolic rate in the Stabilizing High Gain condition was 9% lower

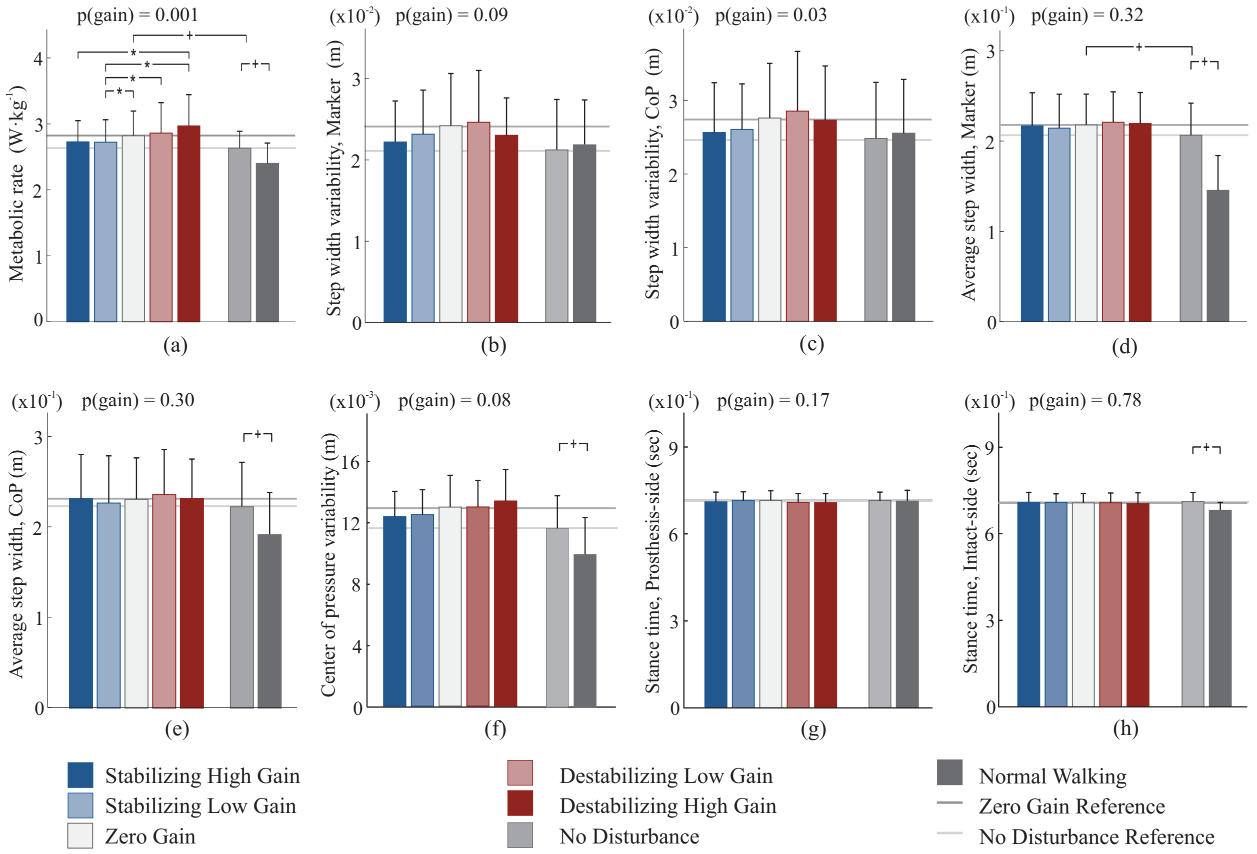

Figure A2: **Measures of balance-related effort without the distraction task.** (**a**) Metabolic rate was reduced by Stabilizing control conditions. (**b**) Step width variability based on foot markers tended to be lower with Stabilizing control. (**c**) Step width variability based on Center of Pressure (CoP) was reduced by Stabilizing gains. (**d**) Average step width based on foot markers was not affected by control gain. (**e**) Average step width based on Center of Pressure (CoP) was not affected by control gain. (**f**) Within-step center of pressure variability tended to be lower with Stabilizing control. (**g**) Prosthesis-side stance time was unchanged across conditions. (**h**) Intact-side stance time was unchanged by control gain. Blue bars correspond to Stabilizing control conditions, white bars to the Zero Gain condition, and red bars to Destabilizing conditions. Darker blue and red bars correspond to High Gains. Light gray bars correspond to the No Disturbance condition, and dark gray bars correspond to the Normal Walking condition. Asterisks (*) indicate statistical significance among control gain conditions, and pluses (+) indicate statistical significance among baseline conditions.

than in the Destabilizing High Gain condition (p = 0.008). Step width variability measured using center of pressure was affected by control gain (ANOVA, p = 0.03), with Stabilizing conditions resulting in lower variability. A similar trend was observed for step width variability measured using foot markers (ANOVA, p = 0.09). Changes in step width variability showed less statistical significance than those during the distraction task period, perhaps because the added cognitive load of the distraction task made prosthesis control more important. Another possibility is that arm motions were affected by holding the clicker used to complete the distraction task, or that the clicker was not held consistently during the first portion of each trial before the distraction task was applied. Baseline comparisons showed similar trends as with the distraction task; wearing the prosthesis (No Disturbance vs. Normal Walking) increased metabolic rate, average step width and within-step center of pressure variability, while the disturbance (Zero Gain vs. No Disturbance) increased metabolic rate and average step width. Other outcomes were not statistically significant.

3. The effect of randomly changing push-off work on balance-related effort

We tested an additional baseline condition in which push-off work was randomly changed on each step, and measured the same balance-related outcomes both with and without the distraction task (Fig. A3). We hypothesized that if push-off work had a strong effect on balance, changing it randomly would strongly increase balance-related effort for the human. We found that random push-off work increased metabolic rate by about 8% compared to the No Disturbance condition ($p = 0.02$). Random push-off work also increased within-step center of pressure variability ($p = 0.04$) and reduced user preference ($p = 0.007$). Other measures of balance-related effort tended to increase with random push-off work.

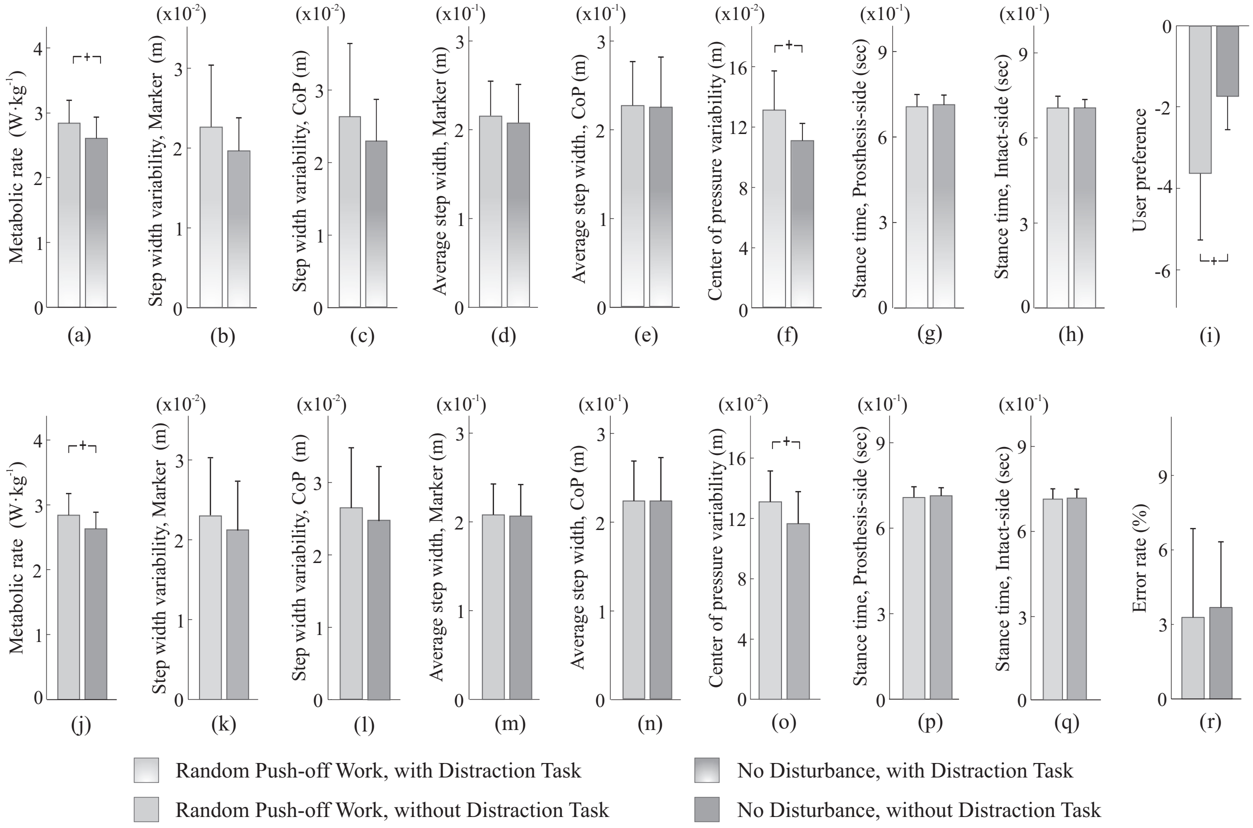

Figure A3: **The effects of random push-off work on balance-related effort.** *Top:* with the distraction task (minutes six to eight). *Bottom:* without the distraction task (minutes four to six). (**a**&**j**) Metabolic rate increased with random push-off work. (**b**&**k**) Step width variability based on foot markers appeared to increase. (**c**&**l**) Step width variability based on Center of Pressure (CoP) appeared to increase. (**d**&**m**) Average step width based on foot markers. (**e**&**n**) Average step width based on Center of Pressure (CoP). (**f**&**o**) Within-step Center of Pressure variability increased with random push-off work. (**g**&**p**) Prosthesis-side stance time. (**h**&**q**) Intact-side stance time. (**i**) User preference decreased with random push-off work. (**r**) Error rate with the distraction task. Light gray bars correspond to the Random Push-off Work condition, and dark gray bars correspond to the No Disturbance condition. Pluses (+) indicate statistical significance (paired t-tests).

4. Average prosthesis push-off work from additional conditions

Average push-off work was unchanged across control gains during the period before the distraction task was applied (Fig. A4(a); p = 0.8). Application of the disturbance (Zero Gain vs. No Disturbance) slightly increased average push-off work. Average push-off work was not changed by the Random Push-off Work condition, with or without the distraction task ($p \geq 0.4$).

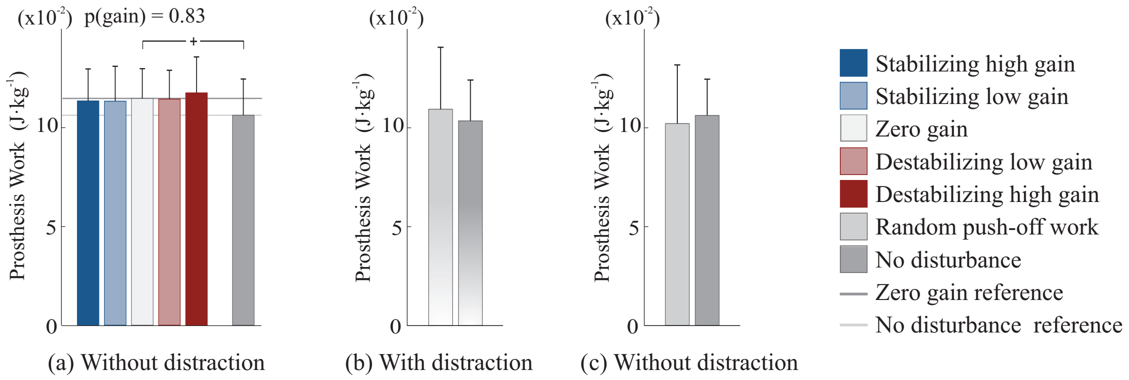

Figure A4: **Average prosthesis push-off work from additional conditions.** (**a**) Push-off work was unchanged across control gains without the distraction task (just as it was unchanged with the distraction task; cf. Fig. 5). (**b&c**) Push-off work was unchanged in the Random Push-off Work condition compared to No Disturbance, with or without the distraction task. Blue bars correspond to Stabilizing control conditions, white bars to the Zero Gain condition, and red bars to Destabilizing conditions. Darker blue and red bars correspond to High Gains. Light gray bars correspond to the Random Push-off Work condition and dark gray bars correspond to the No Disturbance condition. Asterisks (*) indicate statistical significance among control gain conditions, and pluses (+) indicate statistical significance among baseline conditions.

5. Tables of numerical values

Tables A1 and A2 provide the means and standard deviations, respectively, of all balance-related outcomes in the study. In these tables, Stab., Destab., Distract., Disturb., var., avg., CoP, and pref. stand for Stabilizing, Destabilizing, Distraction, Disturbance, variability, average, center of pressure and preference, respectively.

Table A1: Mean values for all balance-related outcomes

Measurement	Distract. task	Stab. High Gain	Stab. Low Gain	Zero Gain	Destab. Low Gain	Destab. High Gain	Random Push-off	No Disturb.	Normal Walking
Metabolic rate	with	2.647	2.732	2.802	2.820	2.885	2.840	2.605	2.333
($W{\cdot}Kg^{-1}$)	without	2.727	2.722	2.820	2.860	2.970	2.840	2.632	2.400
Step width var.	with	0.020	0.021	0.022	0.023	0.022	0.023	0.020	0.020
(marker) (m)	without	0.022	0.023	0.024	0.025	0.023	0.023	0.021	0.022
Step width var.	with	0.023	0.024	0.025	0.026	0.026	0.026	0.023	0.022
(CoP) (m)	without	0.026	0.026	0.028	0.029	0.027	0.026	0.025	0.026
Avg. step width	with	0.216	0.215	0.220	0.217	0.220	0.212	0.207	0.144
(marker) (m)	without	0.217	0.214	0.218	0.221	0.220	0.208	0.207	0.145
Avg. step width	with	0.232	0.228	0.234	0.234	0.235	0.227	0.225	0.193
(CoP) (m)	without	0.232	0.227	0.231	0.236	0.232	0.223	0.223	0.192
Within-step	with	0.011	0.012	0.012	0.012	0.013	0.013	0.011	0.010
CoP var. (m)	without	0.012	0.013	0.013	0.013	0.013	0.013	0.012	0.010
Error rate (%)	with	3.656	3.379	3.933	2.099	3.298	3.279	3.682	3.020
User pref.	-	−3.090	−3.275	−3.625	−3.950	−3.600	−3.650	−1.850	0.000

Table A2: Standard deviations for all balance-related outcomes

Measurement	Distract. task	Stab. High Gain	Stab. Low Gain	Zero Gain	Destab. Low Gain	Destab. High Gain	Random Push-off	No Disturb.	Normal Walking
Metabolic rate	with	0.328	0.387	0.373	0.459	0.479	0.353	0.329	0.269
($W{\cdot}Kg^{-1}$)	without	0.321	0.340	0.376	0.462	0.474	0.333	0.255	0.310
Step width var.	with	0.005	0.005	0.005	0.004	0.005	0.008	0.004	0.003
(marker) (m)	without	0.005	0.005	0.006	0.006	0.005	0.007	0.006	0.005
Step width var.	with	0.006	0.006	0.006	0.006	0.008	0.010	0.006	0.004
(CoP) (m)	without	0.007	0.006	0.007	0.008	0.007	0.008	0.008	0.007
Avg. step width	with	0.040	0.042	0.041	0.036	0.038	0.039	0.043	0.045
(marker) (m)	without	0.037	0.038	0.034	0.034	0.034	0.035	0.035	0.038
Avg. step width	with	0.054	0.057	0.053	0.051	0.050	0.049	0.057	0.054
(CoP) (m)	without	0.049	0.052	0.046	0.050	0.044	0.045	0.049	0.046
Within-step CoP	with	0.002	0.002	0.002	0.001	0.003	0.003	0.001	0.002
var. (m)	without	0.002	0.002	0.002	0.002	0.002	0.002	0.002	0.002
Error rate (%)	with	5.192	3.822	5.111	2.349	4.336	3.577	2.641	2.389
User pref.	-	0.896	1.742	1.737	1.571	1.792	1.616	0.755	0.000

6. Tables of results of statistical analysis of control on balance-related outcomes

The results of repeated measures ANOVA tests for an effect of control gain on balance-related outcomes are presented in Table A3. The results of follow-up paired t-tests between controller conditions, only among outcomes that showed a significant relationship, are presented in Table A4. Asterisks ($*$) denote statistical significance significance ($\alpha < 0.05$).

Table A3: Results of repeated measures ANOVA tests for an effect of control gain

Measure	Distract. task	ANOVA result
Metabolic rate	with	0.005*
	without	0.001*
Step width var. (marker)	with	0.030*
	without	0.091
Step width var. (CoP)	with	0.049*
	without	0.030*
Avg. step width (marker)	with	0.240
	without	0.320
Avg. step width (CoP)	with	0.390
	without	0.300
Within-step CoP var.	with	0.075
	without	0.074
Error rate	with	0.740
User pref.	-	0.449

Table A4: Results of paired t-tests for condition-wise differences among significant outcomes

Conditions Compared		Metabolic rate		Step width var. (marker)		Step width var. (CoP)	
		with distract.	without	with distract.	without	with distract.	without
Zero Gain	Stab. High	0.003*	0.070	0.027*	-	0.009*	0.094
Zero Gain	Stab. Low	0.058	0.018*	0.186	-	0.091	0.234
Zero Gain	Destab. Low	0.802	0.363	0.636	-	0.912	0.586
Zero Gain	Destab. High	0.243	0.053	0.553	-	0.592	0.808
Stab. High	Stab. Low	0.039*	0.911	0.136	-	0.068	0.659
Stab. High	Destab. Low	0.020*	0.063	0.015*	-	0.046*	0.098
Stab. High	Destab. High	0.021*	0.008*	0.055	-	0.030*	0.266
Stab. Low	Destab. Low	0.118	0.049*	0.135	-	0.203	0.082
Stab. Low	Destab. High	0.079	0.011*	0.440	-	0.126	0.272
Destab. Low	Destab. High	0.323	0.141	0.202	-	0.975	0.181

7. Table of results of statistical analysis of baseline conditions

The results of paired t-tests for differences between baseline conditions are presented in Table A5. Asterisks (∗) denote statistical significance significance ($\alpha < 0.05$).

Table A5: Results of paired t-tests comparing balance-related outcomes in baseline conditions

Measure	Distraction task	Random Push-off vs. No Disturbance	Zero Gain vs. No Disturbance	Normal Walking vs. No Disturbance
Metabolic rate	with	0.016*	0.011*	0.001*
	without	0.017*	0.028*	0.008*
Step width var. (marker)	with	0.077	0.114	0.875
	without	0.217	0.058	0.579
Step width var. (CoP)	with	0.109	0.156	0.598
	without	0.094	0.130	0.574
Avg. step width (marker)	with	0.330	0.009*	0.000*
	without	0.630	0.009*	0.000*
Avg. step width (CoP)	with	0.764	0.1330	0.001*
	without	0.993	0.184	0.001*
Within-step CoP var.	with	0.046*	0.102	0.084
	without	0.041*	0.184	0.020*
Error rate	with	0.736	0.513	0.621
User pref.	-	0.007*	0.001*	0.000*
